# Diagnostic prediction of gastrointestinal graft-versus-host disease based on a clinical- CT- signs nomogram model

**DOI:** 10.1186/s13244-024-01654-3

**Published:** 2024-03-22

**Authors:** Qing Feng, Fengming Xu, Kaiming Guan, Tao Li, Jing Sheng, Wei Zhong, Haohua Wu, Bing Li, Peng Peng

**Affiliations:** 1 Department of Radiology, The First Affiliated Hospital of Guangxi Medical University, Shuangyong Road, Nanning, 530021 Guangxi Province China; 2 Department of Radiology, Liuzhou Workers’ Hospital, Heping Road, Liuzhou, 545005 Guangxi Province China; 3 Department of Radiology, Liuzhou People’s Hospital, Guangchang Road, Liuzhou, 545000 Guangxi Province China; 4 NHC Key Laboratory of Thalassemia Medicine, Nanning, 530021 Guangxi Province China

**Keywords:** Clinical CT signs, Diagnostic, Gastrointestinal graft-versus-host disease, Nomogram

## Abstract

**Objective:**

Gastrointestinal graft-versus-host disease (GI-GVHD) is one of the complications that can easily occur after hematopoietic stem cell transplantation (HSCT). Timely diagnosis and treatment are pivotal factors that greatly influence the prognosis of patients. However, the current diagnostic method lacks adequate non-invasive diagnostic tools.

**Methods:**

A total of 190 patients who suspected GI-GVHD were retrospectively included and divided into training set (*n* = 114) and testing set (*n* = 76) according to their discharge time. Least absolute shrinkage and selection operator (LASSO) regression was used to screen for clinically independent predictors. Based on the logistic regression results, both computed tomography (CT) signs and clinically independent predictors were integrated in order to build the nomogram, while the testing set was verified independently. The receiver operating characteristic (ROC), area under the curve (AUC), decision curve, and clinical impact curve were used to measure the accuracy of prediction, clinical net benefit, and consistency of diagnostic factors.

**Results:**

Four key factors, including II-IV acute graft-versus-host disease (aGVHD), the circular target sign, multifocal intestinal inflammation, and an increased in total bilirubin, were identified. The combined model, which was constructed from CT signs and clinical factors, showed higher predictive performances. The AUC, sensitivity, and specificity of the training set were 0.867, 0.787, and 0.811, respectively. Decision curve analysis (DCA), net reclassification improvement (NRI), and integrated discrimination improvement (IDI) showed that the developed model exhibited a better prediction accuracy than the others.

**Conclusions:**

This combined model facilitates timely diagnosis and treatment and subsequently improves survival and overall outcomes in patients with GI-GVHD.

**Critical relevance statement:**

GI-GVHD is one of the complications that can easily occur after HSCT. However, the current diagnostic approach lacks adequate non-invasive diagnostic methods. This non-invasive combined model facilitates timely treatment and subsequently improves patients with GI-GVHD survival and overall outcomes.

**Key points:**

• There is currently lacking of non-invasive diagnostic methods for GI-GVHD.

• Four clinical CT signs are the independent predictors for GI-GVHD.

• Association between the CT signs with clinical factors may improve the diagnostic performance of GI-GVHD.

**Graphical Abstract:**

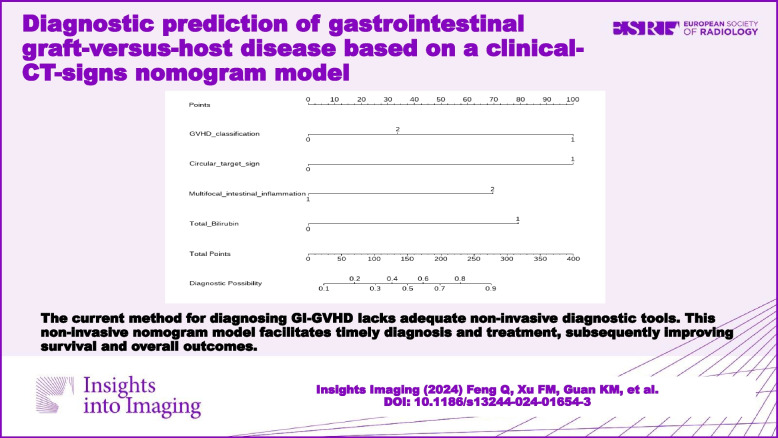

**Supplementary Information:**

The online version contains supplementary material available at 10.1186/s13244-024-01654-3.

## Introduction

Hematopoietic stem cell transplantation (HSCT) helps counteract the effects of tumor and disease, and thus, it is essential for the management of several life-threatening hematological diseases [1, 2]. Graft-versus-host disease (GVHD) can occur after HSCT, and is an immune response resulting from the interaction between donor and recipient cells. The skin, gastrointestinal tract, and liver are the primary organs affected during this condition [3]. Gastrointestinal graft-versus-host disease (GI-GVHD) is observed in more than 60% of GVHD cases [4]. In addition to the increased risk of infection, HSCT also elevates the mortality risk by being a risk factor for GI-GVHD [5]. GI-GVHD cases can present with non-specific gastrointestinal symptoms such as nausea, vomiting, dysphagia, diarrhea, and gastrointestinal bleeding [6, 7]. However, the diagnosis of GI-GVHD can be challenging due to the presence of comorbid conditions such as infection and drug toxicity [8]. Timely diagnosis and treatment of GI-GVHD are of paramount importance to achieving favorable clinical outcomes for patients. Treatment entails implementing immunosuppressive therapy tailored to the clinical grading of GVHD. Although endoscopy and biopsy serve as gold standards for the diagnosis of GI-GVHD, their findings can be non-specific. In addition, these techniques only allow observation of intra-intestinal changes, limiting the detection of extra-intestinal findings [9]. These procedures may also predispose the patients to hemorrhage, another complication of GVHD [10].

In this case, imaging can play an important role in diagnosing GI-GVHD. However, there has been a scarcity of research specifically dedicated to this topic. The scant published studies have predominantly relied on ultrasound, positron emission tomography (PET) scans, and, more recently, magnetic resonance imaging (MRI) techniques [9, 11, 12]. None of these studies have addressed GI-GVHD occurring at the stage of chronic GVHD (cGVHD). Furthermore, prior studies have been hampered by an inadequate volume of data, making it challenging to construct a comprehensive diagnostic model for GI-GVHD. The development of a non-invasive predictive diagnostic model in this study fills this gap and provides a valuable tool for timely diagnosis and treatment of patients at risk of GI-GVHD. This model can help to guide subsequent treatment decisions, leading to early detection and intervention, which in turn can significantly improve patient prognosis.

By constructing a model based on clinical CT signs, this research tackles a significant challenge in managing GI-GVHD and introduces a novel non-invasive approach for a more accurate and effective diagnosis.

## Methods

### Patients

**Fig. 1:**
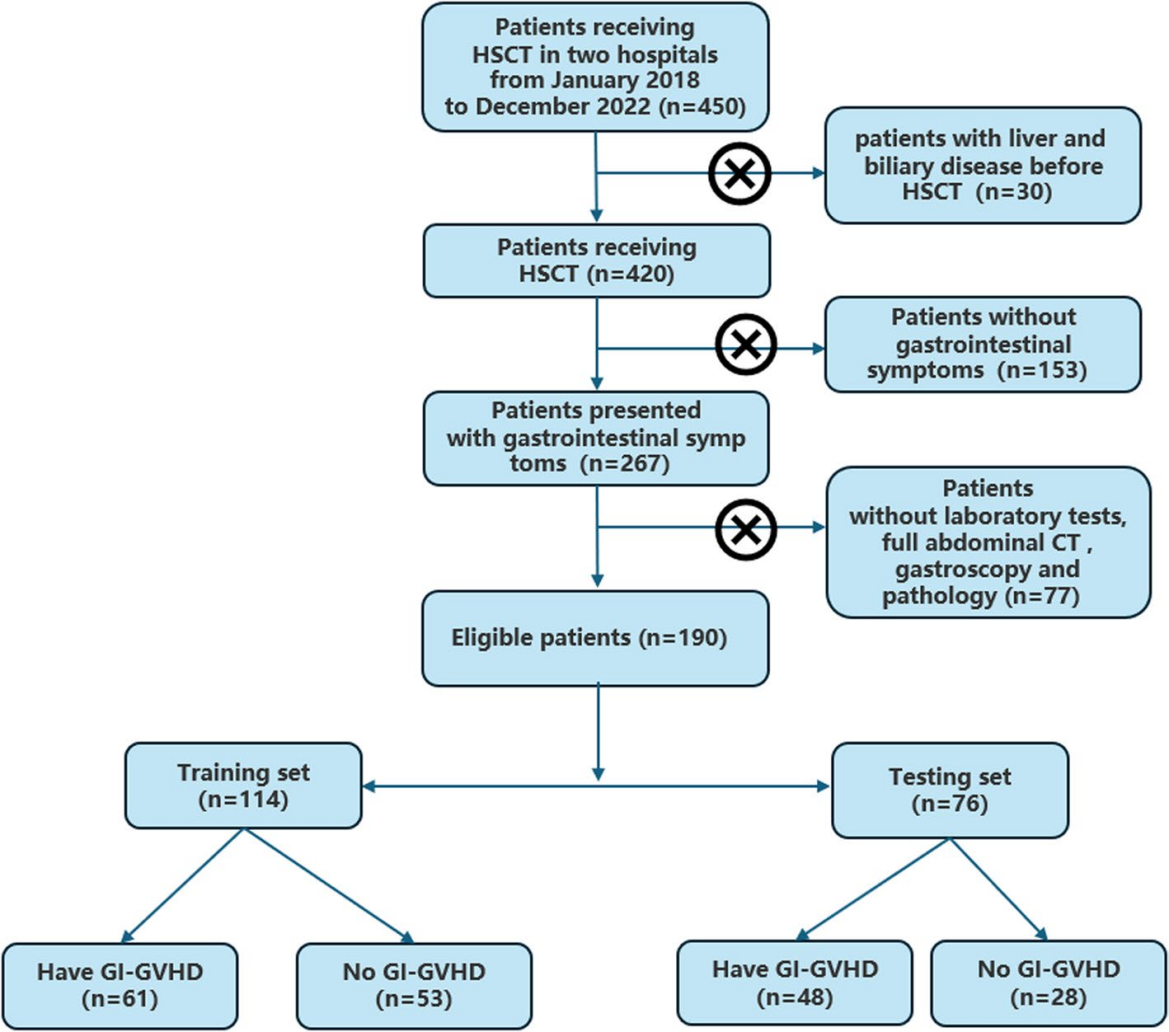
Flow chart of patient screening

Ethical approval was obtained from the institutional review boards of two centers, and patient consent was waived for this retrospective analysis. The ethical numbers are no.2023-E112-01 and no. LW2023016. A total of 450 patients who received HSCT were recruited from two centers between January 2018 and December 2022. Of these, 267 presented with gastrointestinal symptoms and 190 underwent laboratory tests, full abdominal CT, gastroscopy, and pathology. The patients were divided into a training set (*n* = 114) and a testing set (*n* = 76) according to their discharge time (Fig. 1).

The inclusion criteria of this study were as follows: (1) patients who underwent HSCT, (2) patients who had undergone CT-enhanced examinations of the upper and lower abdomen and pelvis, (3) the time interval between the CT examination and gastroscopy was less than 2 weeks, (4) the time interval between the CT examination and laboratory examination was less than 3 days, and (5) the time interval between clinical staging and the CT examination was less than 2 weeks. The exclusion criteria were as follows: (1) patients with fecal culture showing bacteria or fungi, (2) patients diagnosed with biliary and liver disease before HSCT, (3) patients who did not undergo gastroenteroscopy and laboratory tests.

### Laboratory examination and CT examination

In this study, we collected laboratory parameters, as detailed in Additional file 1 (electronic supplementary material).

All CT images were retrieved from the picture archiving and communication system (PACS) for further analysis. The CT image extraction is described in Additional file 2.

### CT image analysis

**Fig. 2:**
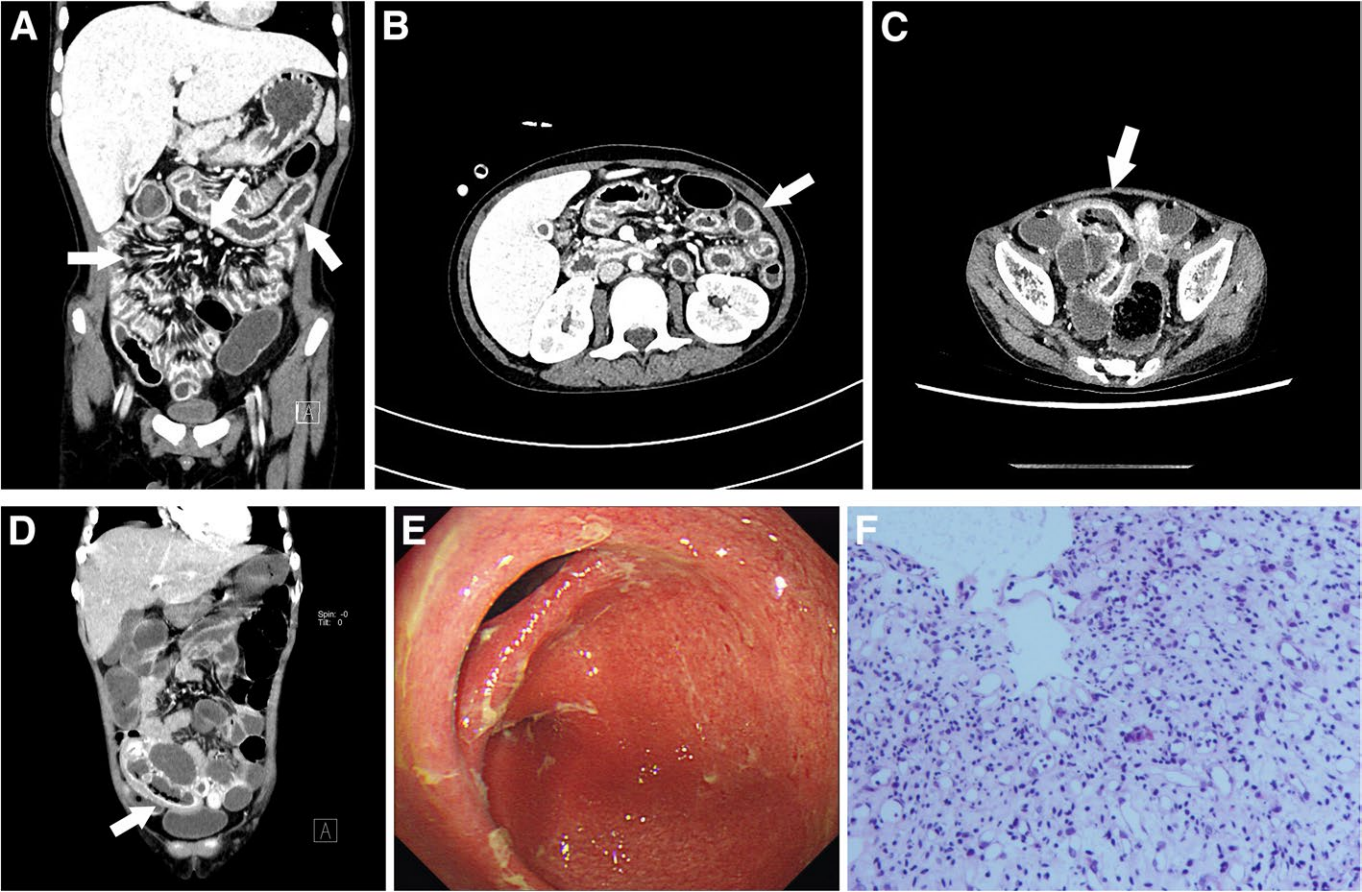
**A**, **B** The patient is a 22-year-old female diagnosed with acute myeloid leukemia, experiencing aGVHD with GI-GVHD. The enhanced CT venous images of the abdomen, as shown in both coronal (**A**) and axial (**B**) views, reveal several notable findings: (a) multifocal inflammation in the small bowel and colon is evident, with significant mucosal enhancement (indicated by the thick arrow on the left in both images). (b) The circular target sign is observable (indicated by the thick arrow in image (**B**)). (c) There is marked edema and comb sign changes in the mesentery (indicated by the arrow on the right in image (**A**)). (d) Small lymph node hyperplasia is visible around the mesentery (indicated by the arrow in the middle in image (**A**)). **C**, **D** The patient is an 11-year-old male with thalassemia and aGVHD affecting the gastrointestinal tract. The enhanced CT venous images of the abdomen, displayed in both axial (**C**) and coronal (**D**) views, with the following observations: (a) in image (**C**), there is the thickening of edema in the wall of the sigmoid colon with pronounced abnormal mucosal enhancement (indicated by the thick arrow). (b) Image **D** shows dilatation and pneumatization of the left colon. Additionally, there are multiple small submucosal air sac formations (also marked by the thick arrow). **E** The gastrointestinal micrograph displays significant congestion and edema in the mucosa of both of the large intestine and terminal ileum. Additionally, there is diffuse flushing and impaired peristalsis. **F** The pathology image at × 100 magnification reveals congestion and edema in the mucosa of the sigmoid colon. Notably, there is the formation of granulation tissue, along with hyperplasia of capillary and fibrous connective tissues, and the intestinal mucosa without epithelial covering

Quantitative imaging measurements included the following: (1) assessment of multifocal intestinal inflammation, segmental wall hyperenhancement (increased attenuation of contrast-enhanced scans of uncontracted segments compared to nearby normal small bowel segments), division of the GI tract into 10 segments (stomach, duodenum, jejunum, ileum, cecum, ascending colon, transverse colon, descending colon, sigmoid colon, and rectum), and counting the number of involved bowel collaterals (multifocal intestinal wall inflammation was defined as the involvement of ≥ 3 groups of bowel collaterals) (Fig. 2A, B); (2) intestinal wall thickening (measuring the thickest part of the most distended segment or the most severe inflammatory site): grading of gastric wall thickening was categorized as normal (< 4 mm), mild (4–6 mm), moderate (6–9 mm), and severe (> 9 mm), grading of small bowel wall thickening was categorized as normal (< 2 mm), mild (2–3 mm), moderate (3–5 mm), and severe (> 5 mm), and grading of colonic wall thickening was categorized as normal (< 5 mm), mild (5–7 mm), moderate (7–10 mm), and severe (> 10 mm) [13]; as shown in Fig. 2C, the case exhibited severe thickening of the sigmoid colon; (3) the circular target sign was defined as a bilaminar shape of the intestinal wall based on a high degree of mucosal enhancement and a reduced intramural attenuation [14] (Fig. 2B); (4) small submucosal gas sacs: three or more small unfused sac-like gas shadows visible under the highly enhanced mucosa (Fig. 2D); (5) the comb sign: the number of straight vessels with an ROI of 1 cm^2^ counted on the reconstructed coronal image; specifically, comb sign changes were defined as the presence of ≥ 5 [14] (Fig. 2A); (6) enlarged peri-mesenteric lymph nodes. We measured the short-axis diameter of the most enlarged lymph nodes and defined enlargement as > 5 mm in diameter or > 3 in number [15] (Fig. 2A). Two radiologists (J.S. and W.Z.), with 5 years and 8 years of experience, respectively, in diagnostic abdominal imaging, analyzed the CT images. A radiologist with 20 years of experience in diagnostic abdominal imaging reviewed all images. In the case of any discrepancy, the radiologists were asked to review the bowel images of the area of interest, and consistent results were used for further statistical analysis. To ensure the reliability and reproducibility of the extracted features, a test-retest analysis was conducted by the same radiologist. This involved performing segmentations on 30 randomly selected patients (*n* = 30), and the second evaluation was conducted one month after the initial assessment. An interclass correlation coefficient (ICC) greater than 0.80 was set as the benchmark for excellent reliability. Features with low intra-observer agreement, as indicated by the ICC below this threshold, were subsequently excluded from the study.

### Gastroscopy and pathology criteria

Gastroscopic images (Fig. 2E) of GI-GVHD were assessed by gastroenterologists at both centers based on the patient's clinical history [7]. Pathologists at both centers made the diagnosis of GI-GVHD in accordance with the National Institutes of Health (NIH) consensus guidelines: the threshold for minimal histological features was ≥ 1 intraepithelial apoptosis or multiple apoptoses in the crypt/glandular body per biopsy [16]. We found intraepithelial crypt cell necrosis and apoptosis as well as many proliferating capillaries, granulation tissues, and fibrous connective tissues among the positive cases [17, 18] (Fig. 2F). In compliance with the NIH guidelines, a minimum of three consecutive sections were analyzed to confirm a positive case.

### Clinical GVHD diagnostic criteria

GI-GVHD is included in the consensus or modified Glucksberg grading system. Grade I gastrointestinal symptoms is exclusively present in grade II acute GVHD (aGVHD) [19]. The clinical grading of GVHD is determined by evaluating its impact on the skin, liver, and intestines, following the relevant guidelines [11].

### Development of prediction models and nomogram

We conducted a statistical analysis on the data, specifically employing a univariate logistic regression. We initially screened for risk predictors using LASSO regression. Subsequently, we developed independent prediction models for GI-GVHD using both the CT-signs and clinical risk predictors in the training dataset. We then validated these models in a separate testing dataset. To evaluate the performance of these models, we employed the receiver operating characteristic (ROC) and calculated the area under the curve (AUC), specificity, and sensitivity. We compared the performance of three different models: one based solely on clinical features (ModA), another based solely on the CT signs (ModB), and a combined model (ModC). This comparison was done using the ROC analysis. Furthermore, we constructed a visual representation of the combined model as a nomogram using logistic regression, in order to enhance its practical applicability in a clinical setting. To assess the nomogram’s performance, we utilized a calibration curve. Additionally, we conducted a decision curve analysis (DCA) to evaluate the clinical utility of the nomogram, specifically calculating the net gain within a defined range of thresholds.

### Statistical analysis

All statistical analyses were performed using the SPSS version 26.0 software (IBM SPSS Statistical Windows, version 25.0. Armonk, NY: IBM Corp.) and R version 4.0.4 (https://www.r-project.org/). The normality of the data was assessed using the Shapiro-Wilk test. The non-normally distributed continuous variables are summarized as median with the interquartile range of 25–75%. Between-group comparisons were made using Student’s *t*-test or Kruskal-Wallis rank sum test, whenever appropriate. Categorical variables are summarized as frequencies (%), and the differences between groups were tested using the chi-square test. All statistical tests were of two-tailed, and *p* < 0.05 was considered as statistically significant.

## Results

### Clinical characteristics

The final cohort study included 190 patients through pathological tissue examination, comprising of 93 (48.9%) males and 97 (51.1%) females. The ages of the patients ranged from 4 to 65 years, with a median age of 14 years (range = 11–32 years). The incidence of GI-GVHD was evenly distributed between the two groups, with 63.2% in the training set and 53.5% in the testing set (*p* = 0.243). Additionally, there were no significant differences in other clinical factors and laboratory tests between the two datasets (Table 1).

**Table 1:** Comparing the clinical characteristics of the two groups (*n*)

Characteristics	Total (*N* = 190)	Training set (*N* = 114)	Testing set (*N* = 76)	*p*
Sex (%)				0.615
Male	93 (48.9)	58 (50.9)	35 (46.1)	
Female	97 (51.1)	56 (49.1)	41 (53.9)	
Age, years (range)	14.0 (11.0–32.0)	15.0 (11.0–31.8)	14.0 (10.0–32.2)	0.711
Diseases of transplantation (%)				0.952
Thalassemia	93 (48.9)	53 (46.5)	40 (52.6)	
Leukemia	86 (45.3)	54 (47.4)	32 (42.2)	
Aplastic anemia	5 (2.6)	3 (2.5)	2 (2.6)	
Myelodysplastic syndrome	4 (2.2)	2 (1.8)	2 (2.6)	
Myeloma	1 (0.5)	1 (0.9)	0	
Hemophagocytic syndrome	1 (0.5)	1 (0.9)	0	
Sources of transplantation (%)				0.833
Unrelated donor	113 (59.5)	69 (60.5)	44 (57.9)	
Related donor	77 (40.5)	45 (39.5)	32 (42.1)	
GVHD classification (%)				0.989
No	44 (23.1)	26 (22.8)	18 (23.7)	
II-IVaGVHD	86 (45.3)	52 (45.6)	34 (44.7)	
cGVHD	60 (31.6)	36 (31.6)	24 (31.6)	
GI-GVHD (%)				0.243
No	81 (42.6)	53 (46.5)	28 (36.8)	
Have	109 (57.4)	61 (53.5)	48 (63.2)	
Bowel wall thickening (%)				0.597
Severe	87 (45.8)	51 (44.7)	36 (47.4)	
Moderate	48 (25.3)	27 (23.7)	21 (27.6)	
Mild	55 (28.9)	36 (31.6)	19 (25.0)	
Circular target sign (%)				0.053
No	105 (55.3)	70 (61.4)	35 (46.1)	
Have	85 (44.7)	44 (38.6)	41 (53.9)	
Pneumatosis cystoides intestinalis (%)				0.164
No	98 (51.6)	64 (56.1)	34 (44.7)	
Have	92 (48.4)	50 (43.9)	42 (55.3)	
Multifocal intestinal inflammation (%)				0.533
Single	101 (57.4)	58 (50.9)	43(56.6)	
Multiple	89 (46.8)	56 (49.1)	33 (43.4)	
Mesangial lymph node hyperplasia (%)				0.650
No	75 (39.5)	43 (37.7)	32 (42.1)	
Have	115 (60.5)	71 (62.3)	44 (57.9)	
Edema of the mesentery (%)				0.244
No	63 (33.2)	42 (36.8)	21 (27.6)	
Have	127 (66.8)	72 (63.2)	55 (72.4)	
CRP, mg/L (range)	4.5 (2.8–31.8)	8.2 (3.0–34.2)	3.7 (2.2–27.6)	0.076
Total bilirubin, μmol/L (range)	23.9 (13.3–41.8)	21.1 (12.7–39.9)	26.3 (16.1–42.2)	0.221

### Univariate and multivariate binary logistic regression analyses of the diagnostic factors in GI-GVHD

**Fig. 3:**
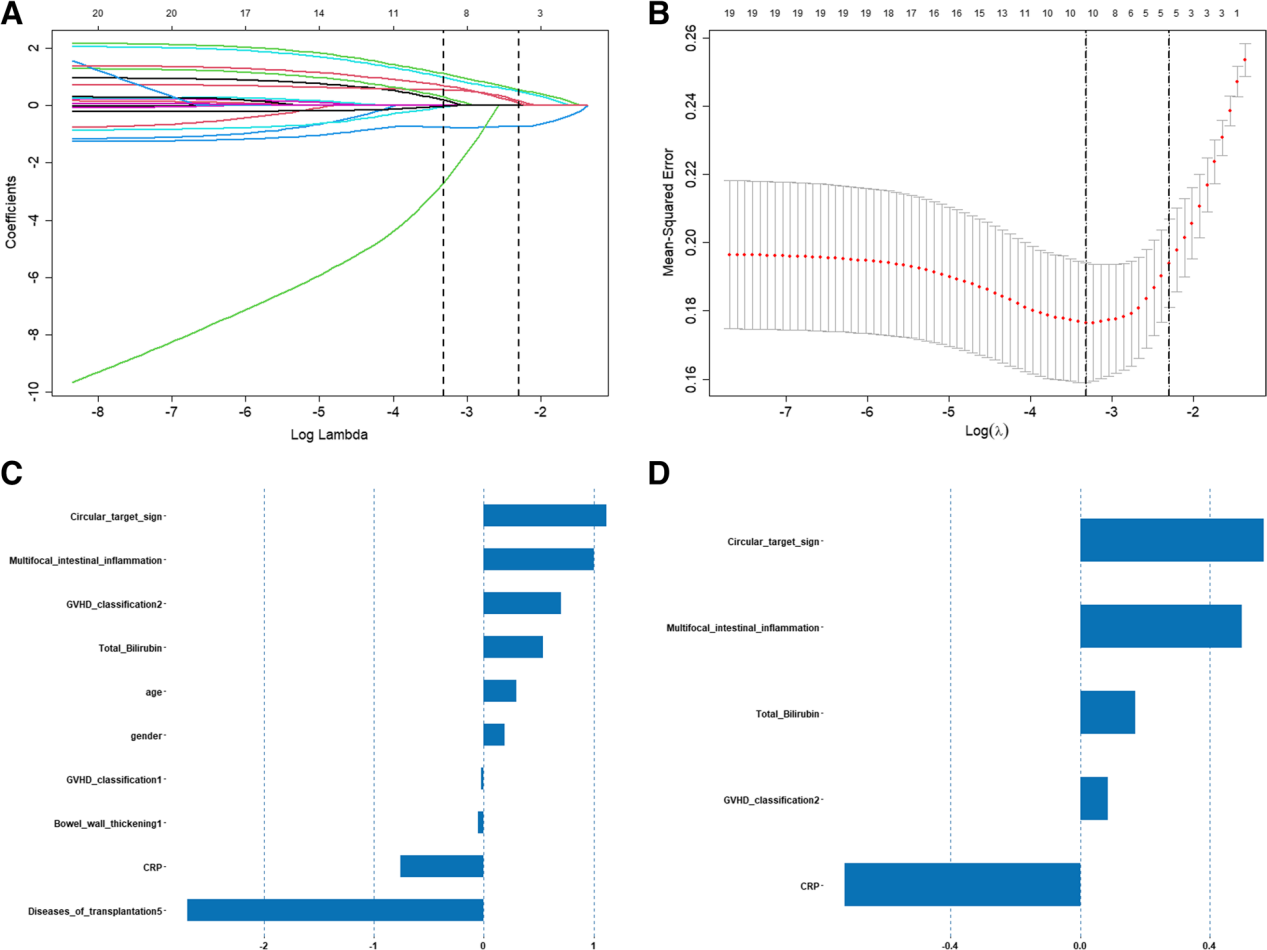
Clinical- CT- signs selection using the least absolute shrinkage and selection operator (LASSO) regression model. Clinical- CT- signs selection using LASSO regression model. **A** Tuning parameter (*λ*) selection in LASSO model with tenfold cross-validation via minimum criterion. The optimal values of the LASSO tuning parameter (*λ*) are indicated by the dotted vertical lines. **B** LASSO coefficient profiles of the 19 clinical CT signs. A coefficient profiles plot was produced versus the log (*λ*) sequence. The dotted vertical line was drawn at the value selected using the tenfold cross-validation, *p* < 0.05 as the inclusion criteria, and the selected *λ* resulted in six nonzero coefficients. **C** The most predictive subset of the feature was chosen, and the corresponding coefficients were evaluated in the training cohort

All variables with *p* < 0.05 were included in the multivariate model (Table 2). These features were then subjected to LASSO analysis to obtain the most valuable criteria. Under the minimum criteria via tenfold cross-validation, eventually, the circular target sign, multifocal intestinal inflammation, II-IV aGVHD, and an increased Tbil were selected to construct the radiomics signature (Fig. 3A, B). The contribution of the radiomics signature is shown in Fig. 3C.

**Table 2:** Univariate and multifactor logistic regression analysis of GI-GVHD diagnostic factors

	**Univariate analysis**		**Multivariate analysis**	
**Characteristics**	**HR (95%) Cl**	***p*-value**	**HR (95%) CI**	***p*-value**
Sex	1.537 (0.735–3.248)	0.255		
Age	1.718 (0.797–3.782)	0.171		
Sources of transplantation				
Thalassemia	0.736 (0.344–1.563)	0.425		
Leukemia	0.767 (0.356–1.64)	0.494		
Aplastic anemia	1.533 (0.139–34.19)	0.734		
Myelodysplastic syndrome	0.767 (0.029–20.08)	0.854		
Myeloma	0 (NA–8.120)	0.991		
Hemophagocytic syndrome	44143 (0–NA)	0.992		
II-IVaGVHD	7.5 (2.658–23.9)	< 0.001	4.39 (1.061–20.86)	0.049
cGVHD	3.725 (1.261–12.21)	0.022	1.423 (0.293–7.132)	0.66
Edema of the mesentery	1.455 (0.678–3.145)	0.336		
Mesangial lymph node hyperplasia	0.862 (0.4–1.842)	0.701		
Multifocal intestinal inflammation	7.35 (3.274–17.45)	< 0.001	3.613 (1.197–11.51)	0.025
Pneumatization of the intestinal wall	3.41 (1.58–7.627)	0.002	0.482 (0.116–1.706)	0.281
Circular target sign	10.13 (4.128–27.96)	< 0.001	4.607 (1.37–16.87)	0.016
Bowel wall thickening (moderate)	1.3 (0.51–3.361)	0.583		
Bowel wall thickening (mild)	1.456 (0.618–3.482)	0.392		
CRP	0.104 (0.042–0.239)	< 0.001	0.316 (0.084–1.131)	0.079
Total bilirubin	4.599 (2.072–10.75)	< 0.001	3.396 (1.206–10.09)	0.023

### Development of a clinical CT sign nomogram model for the diagnosis of GI-GVHD

**Fig. 4:**
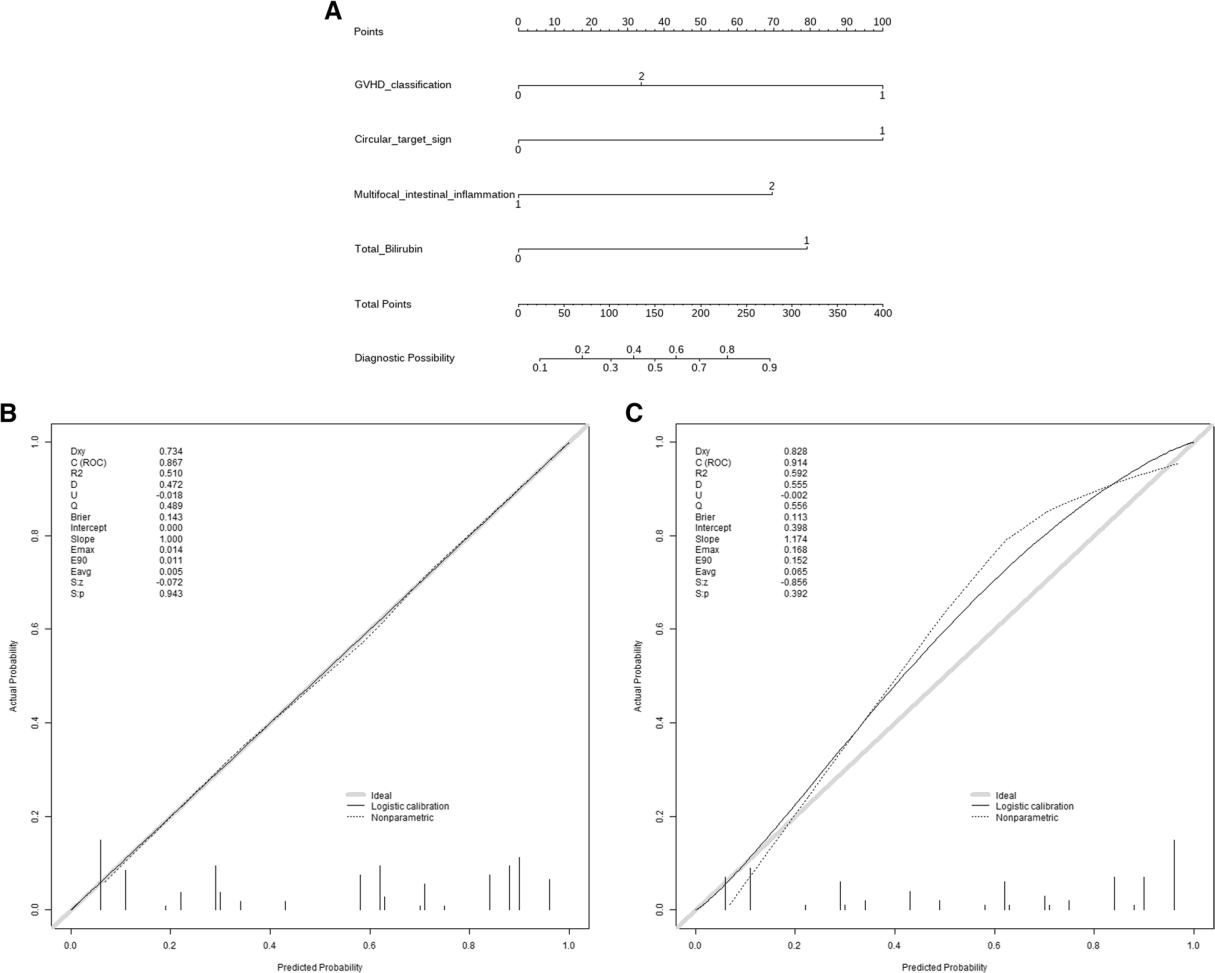
**A** Development of a nomogram based on the clinical CT sign model for diagnosing GI-GVHD. Development of nomogram for predicting the GI-GVHD status. The nomogram was built based on five independent predictors of the training set, including GVHD classification, circular target sign, multifocal intestinal inflammation, and Tbil. In the GVHD classification, 1 represents II-IV aGVHD; 2 represents cGVHD. In the circular target sign, 1 represents have, and 0 represents no. In the multifocal intestinal inflammation, 2 indicates that more than 3 groups of bowel collaterals were involved, and 1 was not reached. In the Tbil, 0 means no increase, and 1 means increase. **B** Calibration curves of the nomogram in the training set. **C** Calibration curves of the nomogram in the testing set

**Fig. 5:**
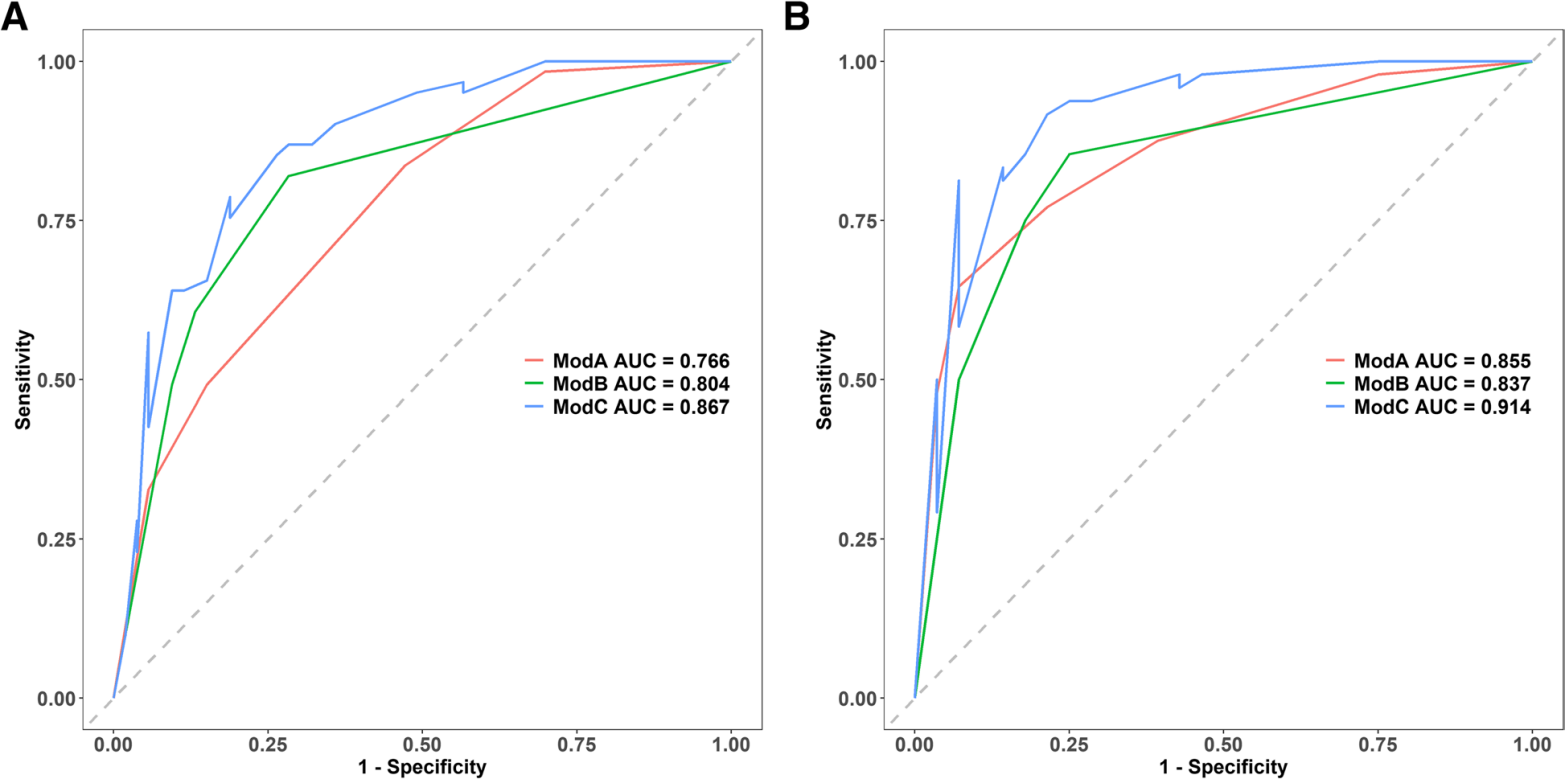
**A** ROC curve for the clinical model (ModA), CT sign model (ModB), and combined model (ModC) in the training set. **B** ROC curves for the clinical model (ModA), CT sign model (ModB), and combined model (ModC) in the test sets (**A** and **B**)

**Fig. 6:**
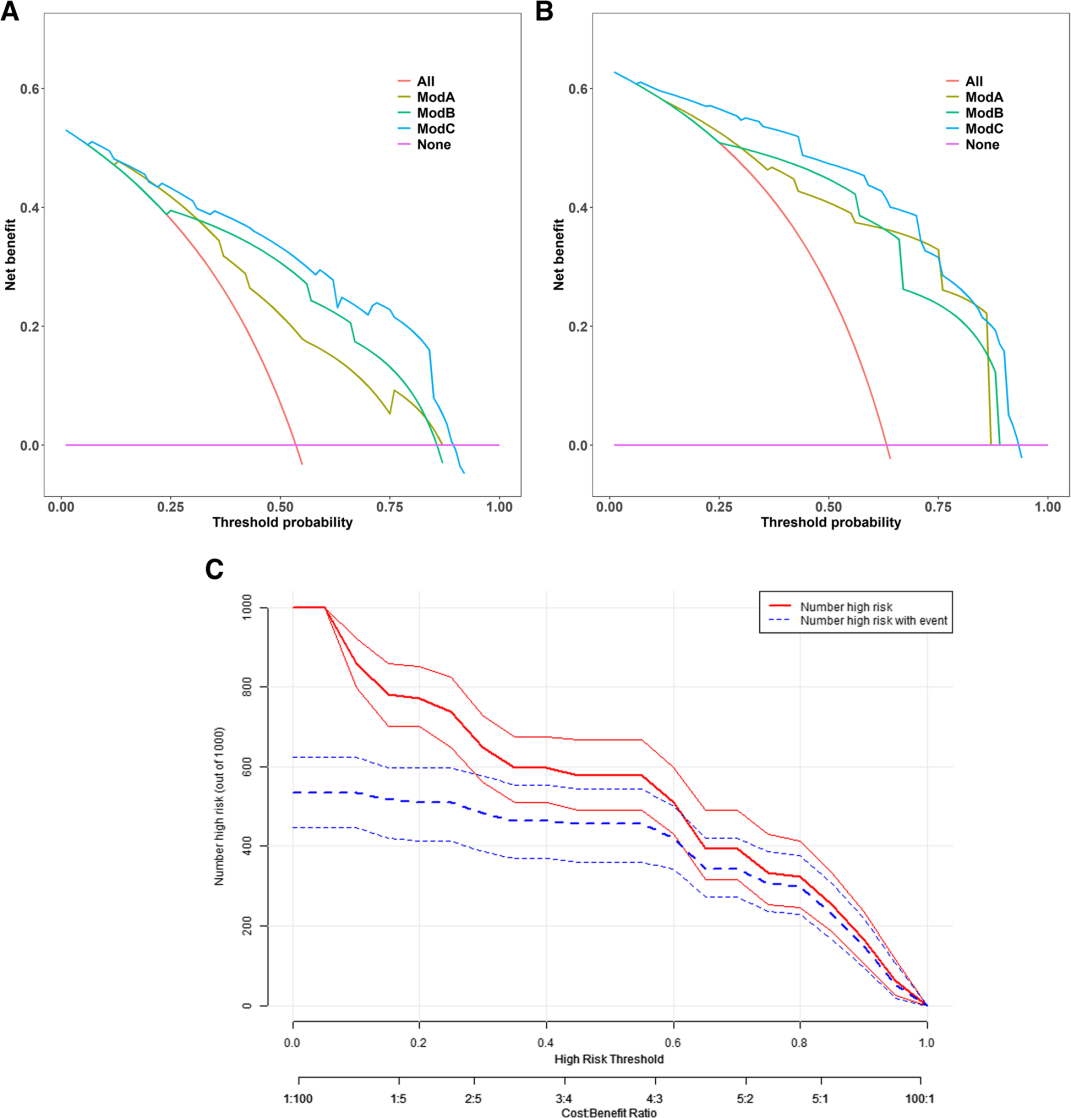
DCA for the clinical model (ModA), CT sign model (ModB), and combined model (ModC) in the training set (**A**) and in the testing set (**B**). **C** Clinical impact curve for the prediction of GI-GVHD

The circular target sign, multifocal intestinal inflammation, II-IVaGVHD, and an elevated Tbil level were integrated into the nomogram (Fig. 4A). The calibration curves displayed excellent agreement between the nomogram's predictions and the actual occurrence of GI-GVHD in both the training and test sets (Fig. 4B, C). The combined nomogram model demonstrated strong predictive performance, with AUCs of 0.867 (95% CI: 0.787–0.811) in the training set and 0.914 (95% CI: 0.812–0.929) in the test set. Figure 5 illustrates a comparison of ROC performance between the clinical model, CT sign model, and combined model in both the training and testing sets; the combined model had higher AUC than other models in training set and testing set. In both datasets, the nomogram outperformed the CT sign model and the clinical model (Table 3), with statistically significant improvements measured by *p-*values for NRI or IDI (Table 4). The DCA for the various models in both the training and test sets is presented (Fig. 6A, B). It is evident that the radiomics nomogram provided greater overall net benefits compared to either the CT sign model or the clinical model. As depicted in the clinical impact curve (Fig. 6C), when the prediction model was employed to stratify risk for a population of 1000 individuals, the two curves closely overlapped, indicating the favorable performance of the predictive model in clinical applications.

**Table 3:** Predictive efficacy of clinical model, CT sign model and combined model

**Dataset**	**AUC**	**SEN**	**SPE**	**PPV**	**NPV**
Clinical train (ModA)	0.766	0.836	0.528	0.671	0.737
Clinical test (ModA)	0.855	0.875	0.607	0.792	0.739
CT signs train (ModB)	0.804	0.82	0.717	0.769	0.776
CT signs test (ModB)	0.837	0.854	0.75	0.854	0.75
Combined train (ModC)	0.867	0.787	0.811	0.828	0.768
Combined test (ModC)	0.914	0.812	0.929	0.951	0.743

**Table 4:** The NRI and IDI of the comparison between clinical model, CT sign model and combined model

	**Training set**				**Testing set**			
	NRI (95% CI)	*p*	IDI (95% CI)	*p*	NRI (95% CI)	*p*	IDI (95% CI)	*p*
Combined model vs clinical model	0.230 (0.071–0.390)	< 0.001	0.187 (0.118–0.257)	< 0.001	0.220 (0.021–0.420)	< 0.001	0.182 (0.103–0.262)	< 0.001
Combined model vs CT sign model	0.716 (0.375–1.056)	< 0.001	0.114 (0.057–0.172)	< 0.001	1.113 (0.727–1.499)	< 0.001	0.189 (0.109–0.268)	< 0.001

## Discussion

According to the existing literature, various non-invasive tests have been proposed for the diagnosis of GI-GVHD. However, each of these tests has certain limitations. Magnetic resonance enterography (MRE) is a time-consuming procedure and may not effectively detect bleeding in the bowel lumen [20]. Ultrasonography is prone to artifacts caused by colonic pneumatization [9]. 18F-FDG-PET CT has been used in some studies on GI-GVHD [11, 21], but hepatic iron overload can lead to the attenuation of correcting the artifacts making it difficult to obtain accurate uptake measurements for the threshold calculations. Moreover, the results can be confounded by medications used among the patients. The significance of this study lies in the fact that the majority of patients who underwent HSCT were children with thalassemia and liver iron overload, making them unsuitable for GI-GVHD assessment using the traditional methods.

The main histological features of GI-GVHD include epithelial apoptosis and degeneration of glandular or crypt cells, with apoptotic cells that contained debris-filled vacuoles. Advanced stages of the disease exhibited crypt cystic dilatation, crypt abscesses, epithelial necrosis, and complete mucosal detachment [17, 22, 23]. These pathological features lead to the congestion and inflammation of the capillary bed, which could be observed endoscopically as mucosal atrophy or detachment, hemorrhage, and congestion [24] (Fig. 4A). However, a significant number of crypt abscesses increases gas production and appear as small submucosal air sacs when it is not fused. After fusion, they cause bowel dilatation and substantial gas accumulation. Brodoefel et al. [25] reported that 94% of the incidence of dilated and pneumatized bowel among GI-GVHD patients and concluded that this manifestation is a key characteristic on CT scans. Although the univariate analysis showed a statistically significant relationship with small intestinal submucosal changes (*p* < 0.05), it was not appropriate to include it in the nomogram.

Previous studies have reported that approximately 60% of GVHD patients displayed mesenteric vascular congestion and edema in the form of comb-like changes [26] (Fig. 2A), including blurring of the surrounding fatty spaces, which are particularly prominent in the thickened bowel wall. However, these signs are non-specific to GI-GVHD and may overlap with other causes of enterocolitis.

Intestinal wall thickening is generally considered as a strong predictor for GI-GVHD [27]. Among patients with GI-GVHD, ileal wall thickening is the most common observation [28], followed by colonic wall thickening. In this study, we also observed severe wall thickening of the sigmoid colon (Fig. 3A). Patients with aGVHD often exhibit oedema in the intestinal wall, resulting in thickening of the wall. On the other hand, patients with cGVHD can develop massive fibrosis in the intestinal wall, leading to the narrowing of the lumen [20], which includes esophageal webs or strictures and, less commonly, segmental small bowel or colonic strictures. These focal stenoses may be accompanied by some upstream dilatation or partial obstruction. Because the intestinal wall behaves differently in the two periods, the final intestinal wall thickening cannot be used as an independent factor for the diagnosis of GI-GVHD in this study.

To account for the potential influence of infection on intestinal wall thickening, the change of CRP was included as one of the factors in the univariate analysis. However, the results of multivariate analysis showed that the change of CRP could not be an independent factor for the diagnosis of GI-GVHD, as the crypt abscess formed by GI-GVHD was combined with a small amount of inflammation. Consistent with previous reports [5], inflammatory parameters did not improve GI-GVHD prediction.

GVHD is one of the most common complications after allogeneic HSCT, with gastrointestinal involvement observed in 74% of aGVHD patients and 30% of cGVHD patients [29]. Therefore, in the multivariate analysis, II-IV aGVHD was identified as one of the independent factors for the diagnosis of GI-GVHD.

Unlike GI-GVHD, neutropenic small bowel colitis is typically limited to the cecum and ascending colon, with occasional involvement of the ileum [14]. In contrast, GI-GVHD can involve both the small bowel and colon, with extensive bowel involvement in a multifocal distribution [30], and strict right colon involvement is not common [31]. In the present study, the majority of patients with GI-GVHD showed multifocal intestinal inflammation in the stomach, small intestine, and colon. Therefore, multifocal bowel involvement was also identified as an independent factor for diagnosis of GI-GVHD.

The circular target sign is a non-specific indicator of intestinal inflammation and is associated with clinically active bowel disease [26]. Shimoni et al. [32] suggested that the incidence of the abnormal “ring target sign” enhancement of the intestinal wall in aGVHD is 16%. However, differences between their findings and the present study may be attributed to different stages of GVHD observed in the cohorts. In cGVHD, the mucosal tissue becomes fibrotic and the circular target sign appears due to the intensification of the intestinal mucosa granuloma [17, 21, 33]; this fibrosis of the intestinal wall leads to luminal narrowing [34]. In our study, we found that the circular target sign was an independent factor in the diagnosis of GI-GVHD.

Additionally, we observed that elevated Tbil was another independent factor in diagnosing GI-GVHD. This is because GI-GVHD is often associated with hepatobiliary involvement [14]. The histological features include biliary epithelial cell apoptosis with cholestasis [35]. Ketelsen et al. [36] also reported a significantly higher rate of common bile duct dilatation in HSCT patients with GI-GVHD compared to those without GI-GVHD. It may be related to the blockage of the bile ducts by biliary sludge. Furthermore, 96% of patients with aGVHD exhibit a significant correlation between bilirubin concentration and the total bile duct diameter. Serum bilirubin levels > 80 mmol/L [31] are an early predictor of mortality in patients with GI-GVHD.

The ability of the model to distinguish between positive and negative cases, as measured by the AUC in the training sets, was 0.867, indicating the predictive model can better distinguish GI-GVHD from non-GI-GVHD. NRI and IDI confirmed the superiority of the predictive models over the other models. The calibration curves also demonstrated excellent consistency between the predicted value and the actual outcome. Furthermore, DCA results revealed that the combined model had more net benefits than those of the clinical model and CT sign model at different threshold calculations. Although our study has limitations and further research with larger prospective studies is needed to fully validate the generalizability of this scoring system, these findings have significant implications. They offer a new diagnostic approach for GI-GVHD, including the identification of key risk factors, validation of model performance, and improving patient prognosis by enabling early and accurate diagnosis and appropriate management.

## Conclusion

Ultimately, these findings may have important implications for clinical practice in managing HSCT and GI-GVHD, which are beneficial to both patients and healthcare providers.

### Supplementary Information

**Additional file 1.** Laboratory Tests of enrolled patients.

**Additional file 2.** CT examination image extraction of enrolled patients.

## Data Availability

The raw data supporting the conclusions of this article will be made available by the authors, without undue reservation.

## Abbreviations

aGVHD: Acute graft-versus-host disease
AUC: Area under the curve
cGVHD: Chronic graft-versus-host disease
CI: Confidence interval
CT: Computed tomography
DCA: Decision curve analysis
GI: Gastrointestinal
GI-GVHD: Gastrointestinal graft-versus-host disease
GVHD: Graft-versus-host disease
HSCT: Hematopoietic stem cell transplantation
ICC: Interclass correlation coefficient
IDI: Integrated discrimination improvement
LASSO: Least absolute shrinkage and selection operator
MRI: Magnetic resonance imaging
NRI: Net reclassification improvement
OR: Odds ratio
PET: Positron emission tomography
ROC: Receiver operating characteristic
Tbil: Total bilirubin
