# Supplementary material for: Diagnostic prediction of gastrointestinal graft-versus-host disease based on a clinical- CT- signs nomogram model
Source: Insights Imaging. 2024 Mar 22;15:84. doi: 10.1186/s13244-024-01654-3 (PMC10959888; doi:10.1186/s13244-024-01654-3)
Supplement: Supplementary file 1 — Additional file 1. Laboratory Tests of enrolled patients. [file 13244_2024_1654_MOESM1_ESM.pdf]

Diagnostic prediction of gastrointestinal graft-versus-host disease based on a clinical-CT-signs nomogram model

ELECTRONIC SUPPLEMENTARY MATERIAL

Additional File 1: Laboratory Tests of enrolled patients

There were no significant differences in laboratory tests between the training and validation set groups. However, total bilirubin (Tbil) was significantly higher and CRP was significantly lower in patients with GI-GVHD when they were included in the univariate analysis (both $p<0.05$), as shown in Table 2. Tbil was expressed in millimoles per liter of serum, and its levels of <21 µmol/l were considered to be within the normal physiological range. CRP was expressed as milligrams per liter of serum, and its levels of <6.0 mg/l were considered to be within the normal physiological range.
