# Supplementary material for: Diagnostic prediction of gastrointestinal graft-versus-host disease based on a clinical- CT- signs nomogram model
Source: Insights Imaging. 2024 Mar 22;15:84. doi: 10.1186/s13244-024-01654-3 (PMC10959888; doi:10.1186/s13244-024-01654-3)
Supplement: Supplementary file 2 — Additional file 2. CT examination image extraction of enrolled patients. [file 13244_2024_1654_MOESM2_ESM.pdf]

Additional File 2: CT examination image extraction of enrolled patients

To prepare the bowel for the CT examination, all patients were instructed to follow a low-fiber/low-residue diet the day before the test and to fast starting from 10 pm. Ten minutes prior to the test, patients were asked to drink 500 ml of warm water to ensure sufficient gastric dilatation. The imaging analysis was conducted using a Siemens Somatom Definition Flash dual-source CT machine from Germany at both hospitals. The specific scan parameters were as follows: a spiral scan speed of 0.7 s/r, pitch of 0.984, detector width of 0.625 mm x 64, and instantaneous high-speed switching between high and low energy (140, 80 kVp) voltages, with a tube current of 360 mA. Enhancement scans were performed using a three-phase dynamic approach with a high-pressure injection of the non-ionic contrast agent iohexol (320 mg I/L) through an antecubital vein at a dose of 1.6 ml/kg. At the beginning of the arterial phase, it was monitored at a CT threshold set at the level of the abdominal aortic trunk, using the Smart Prep technique, with a 150 HU threshold and a 7-second delay after reaching the monitoring threshold. After the arterial phase, there was a 30-second delay before the start of the venous phase, and a 5 mm section thickness after the end of the venous phase. For post-processing, axial image reconstruction was performed, and the images were transferred to an image archiving and communication system for interpretation. The slice thickness was set to 0.6 mm with a reconstruction interval of 0.3 mm. Multiplanar reconstruction and maximum intensity projection techniques were also utilized to assess wall and mesenteric abnormalities.
